# Association between -238 but not -308 polymorphism of Tumor necrosis factor alpha (TNF-alpha)v and unexplained recurrent spontaneous abortion (URSA) in Chinese population

**DOI:** 10.1186/1477-7827-8-114

**Published:** 2010-09-28

**Authors:** Chunmei Liu, Jing Wang, Sirui Zhou, Binbin Wang, Xu Ma

**Affiliations:** 1 National Research Institute for Family Planning, Beijing, 100081 China; 2 Peking Union Medical College, Beijing, China; 3 World Health Organization Collaborating Centre for Research in Human Reproduction, Beijing, China

## Abstract

**Objectives:**

TNF-alpha is a critical cytokine produced by Th1 cells while altered T helper 1 (Th1)-Th2 balance is found crucial for a successful pregnancy.

**Study Design:**

A cohort of 132 Southern Chinese Han RSA patients and 152 controls constituted the subjects of this study. Two functional polymorphisms -308 and -238 of TNF-alpha were studied by association analysis.

**Results:**

lack of association was found in TNF-alpha -308 SNP yet a significant difference was discovered in -238 polymorphism.

**Conclusion:**

This study suggested that TNF-alpha may be a risk factor in Chinese RSA patients. However the ethnic differences may also contribute to the results.

## Introduction

Recurrent spontaneous abortion (RSA) is defined as three or more consecutive pregnancy losses before 22 gestational weeks or the spontaneous abortion of an embryo/fetus weighing less than 500 g and affecting an estimated 1 of every 100 couples preparing to have children [1]. The diagnosis of RSA undergo multiple tests to detect parental chromosomal anomalies, maternal thrombophilic, endocrine, or immunological disorders, yet it has been estimated that the specific cause of RSA remains unknown in 37%-79% of affected women. Over 50% of the RSA cases are classified as idiopathic unexplained recurrent spontaneous abortion (URSA) [1].

Possible candidates include genes regulating the development of increased cellular immunity [2] and altered T helper 1 (Th1)-Th2 balance as well as autoimmune abnormalities, coagulation, angiogenesis, vascular tone, and apoptosis.

Successful pregnancy is dependent on maintaining a fine balance between Th1 and Th2 immunity so forth Th1 and Th2 intracellular cytokine expression is supposed to alter in RSA patients. Over expression of Th1 cytokines is thus associated with RSA in several studies [3]. Infiltration by natural killer (NK) cells and activated macrophages were also detected in resorption sites.

Produced by Th1 cells, cytokine Tumor necrosis factor alpha (TNF-alpha) is a multifunctional proinflammatory cytokine secreted predominantly by monocytes/macrophages that has effects on lipid metabolism, coagulation, insulin resistance, and endothelial function and was proposed as a candidate gene for the pathogenesis of RSA. The evidence was supported by previous studies in which higher serum levels of TNF-alpha were detected in URSM groups [4].

It is suggested that Th1 cytokines trigger thrombotic/inflammatory processes at the maternal uteroplacental blood vessels by activation of vascular endothelial cell procoagulant [5]. The administration of TNF-alpha to normal preganant mice significantly increased fetal resorption.

TNF-alpha was located in 6p21.3 and has several functional sites of polymorphisms. Two common functional polymorphisms in the promoter region of TNF-alpha located at nucleotides -238 (rs361525) and -308 (rs1800629) with respect to the TNF transcriptional start site are all substitutions of adenine for guanine. In vitro studies of these polymorphisms have been shown to increase TNF-α production after lipopolysaccharide stimulation [6]. The objective of the study is to have both the functional SNPs genotyped in order to estimate the relevance between TNF-alpha and URSA in Chinese women.

## Materials and methods

### Subjects

A total of 132 URSA patients and 152 healthy controls were recruited from the Peking Union Medical College, China. All recruited people were Han population and their ancestries were at North China plain area (Beijing, Tianjin and Hebei province). The study was approved by the Ethics Committee of the National Research Institute for Family Planning and informed consent was obtained from all participants. Inclusion criteria were defined as continuous two or more times of the spontaneous loss of pregnancy prior to the 22nd gestational week of pregnancy. All cases were excluded from the study when routine clinical assessments identified the all possible causes for RSA. Controls were individuals of proven fertility, with normal menstrual cycles and ovary morphology, without the history of subfertility treatment.

### DNA analysis

Blood clots were dissolved in TES (100 mM Tris, 100 mM EDTA, 2% SDS). Genomic DNA was extracted from blood clot using QIAamp genomic DNA kits [7].

The *TNF-α *has been mapped to chromosome 6p21.3, the -238 and -308 and the surrounding regions were amplified in all individuals by polymerase chain reaction (PCR) with a pairs of following region-specific primers set, TNF-α-F: 5'-CAAACACAGGCCTCAGGACTC-3', TNF-α-R: 5'-AGGGAGCGTCTGCTGGCTG-3'. These primers were designed by GeneTool V 1.0.0.1 (Launcher Program for BioTools Inc Applications).

PCR products were denatured and annealed to form potential heteroduplexes. Forward products were sequenced using Big Dye Terminator 3.1 chemistry (Applied Biosystems Inc.) with the above primers and run on a 3730xl DNA analyzer (ABI).

### Genetic and statistic analysis

In this study, statistical analyses were carried out using the Statistical Package for Social Sciences version 10.0 (SPSS 10.0). Differences between noncontiguous variables, genotype distribution and allele frequency were tested by chi-square analysis. Significant differences between or among groups was indicated by a P-value < 0.05.

## Result

The average age of the URSA patients was 30.1 ± 4.2 (range from 22 to 40) while in control group it was significantly lower, 28.8 ± 5.7 (range from 16 to 44).

The genotype and allele distributions of the -308 polymorphism in URSA subjects and controls were shown in table 1. Between cases and control groups displayed no significant differences (χ^2 ^= 5.09, df = 2, *P *= 0.078 by genotype; χ^2 ^= 2.61, df = 1, *P *= 0.102 by allele).

**Table 1:** Genotype distribution and relative allele frequencies of -308 polymorphism of *TNF-alpha *gene in Chinese with URSA (n = 132) and controls (n = 152).

Group	No.	Genotype frequency (%)			Allele frequency (%)	
		
		AA	AG	GG	A	G
URSA	132	0 (0)	22 (16.7)	110 (83.3)	22 (8.3)	242 (91.7)
Controls	152	1 (0.6)	13 (8.6)	138 (90.8)	15 (4.9)	289 (95.1)
		χ^2 ^= 5.1, df = 2, *P *= 0.078			χ^2 ^= 2.7, df = 1, *P *= 0.102	

In -238 polymorphism, the AA genotype were not appearance in the present study. The genotype and allele distributions of the -238 polymorphism in URSA subjects and controls were also shown in table 2. Between cases and control groups displayed significant differences (χ^2 ^= 6.86, df = 1, *P *= 0.009 by genotype; χ^2 ^= 6.60, df = 1, *P *= 0.01 by allele). Compared with controls, there was a higher G allele frequency of -238 polymorphism in the RSA cases, 98.5% vs. 94.4%. And the association to URSA reached significance (OR = 4.03 95% CI: 1.30-12.38). Because of the significant differences of ages between case and control groups, we performed the regression analysis to adjust the *P *value by age, according to the result, the association to URSA still reached significance (*P *= 0.016, OR = 3.95, 95% CI: 1.29-12.05 by recessive mode, adjusted by age).

**Table 2:** Genotype distribution and relative allele frequencies of -238 polymorphism of *TNF-alpha *gene in Chinese with URSA (n = 132) and controls (n = 152).

Group	No.	Genotype frequency (%)			Allele frequency (%)	
		
		AA	AG	GG	A	G
URSA	132	0 (0)	4 (3.0)	128 (97.0)	4 (1.5)	260 (98.5)
Controls	152	0 (0)	17 (11.2)	135 (88.8)	17 (5.6)	287 (94.4)
		χ^2 ^= 6.9, df = 2, *P *= 0.009			χ^2 ^= 6.6, df = 1, *P *= 0.010	

We divided the case group according to the different number of times of constitutive abortions. Unfortunately, there weren't significant differences of genotype or allele distribution among groups.

## Discussion

RSA is happening as a heterogeneous condition precipitated by acquired and inherited risk factors. One of them is contributing to Th1 related cytokines encoded genes which proven by the fact that elevated Th1/Th2 cytokine producing CD3(+)/CD4(+) cell ratios were reported in women with a history of recurrent spontaneous abortion (RSA) and multiple implantation failures [8].

TNF-alpha is a potent pleiotropic proinflammatory cytokine that is produced by various type of cells including macrophages, neutrophils, fibroblasts, keratinocytes, NK cells, T and B cells, and tumor cells and affecting growth, differentiation, cellular function and survival of all cells [9].

Disease related to inflammatory factor TNF-alpha includes a variety of category such as tuberculosis [10], Alzheimer's disease [11], and asthma [12]. Nevertheless conflicting studies focus on TNF-alpha polymorphisms and RSA had been published. TNF-alpha -308 polymorphism was reported linked with RSA in one study [13] yet others failed to find the association [14-16].

The present study failed to establish the association between TNF-alpha -308 and URSA in Chinese population. Comparing to healthy controls, although there were no significant differences, the A allele frequency was higher in URSA patients group (4.9% vs. 8.3%, respectively). Furthermore, it should be noticed that the sample size of the present study was larger than that in Costeas's study (132 patients vs. 69 patients) [13], which provided a more sufficient power to detect the potential candidacy of -308 polymorphism and the development of URSA.

Since Chinese Han population was genetically heterogeneous, it should be noticed that patients and controls recruited from the present study were all Chinese Han population and their ancestries were all at North China plain area, the construction of the study population excluded the ethnic interfere to the results of the association analysis. According to the result of the present study, our data clearly demonstrate that G allele of -238 polymorphism possibly be a risk factor of URSA developing in Chinese Han population, which was a conflict result comparing with the previously one [16]. A certain genetic variant may interact with other variants and local environmental influences such that it alters phenotype only in a particular group which suggested an obvious ethnic difference.

There is no strong evidence showing that the TNF-238 polymorphism has a direct effect on gene expression, although studies suggest that this region contains a strong repressor site (-280 to -172) [17]. The TNF-238 polymorphism was within the class III region of the MHC on chromosome 6p. Thus, the TNF-238 polymorphism may be in linkage disequilibrium with a functional polymorphism that impacts TNF production, either within the TNF gene or another gene within the MHC.

To our knowledge, this work firstly established an association between TNF-alpha gene polymorphisms and the development of URSA in Chinese population.

In conclusion, the present study indicated that -308 polymorphism of *TNF-alpha *gene possibly not be a potential risk factor of URSA developing, but patients with URSA has a 3.95-fold higher frequency of -238 GG genotype adjusted by age, which demonstrated the association between -238 polymorphism and the URSA developing in Chinese population.

## Competing interests

The authors declare that they have no competing interests.

## Authors' contributions

CL collected all samples and performed the clinical tests. JW carried out the molecular genetic studies and performed the statistical analysis. SZ drafted the manuscript. BW participated in its design and coordination and helped to draft the manuscript. XM participated in the design of the study. All authors read and approved the final manuscript.
